# Feeding Yellow Worms to Meagre: Effects on Whole-Body Fatty Acid Profile and Hepatic and Intestine Oxidative Status

**DOI:** 10.3390/antiox12051031

**Published:** 2023-04-29

**Authors:** Inês Guerreiro, Carolina Castro, Cláudia R. Serra, Filipe Coutinho, Ana Couto, Helena Peres, Pedro Pousão-Ferreira, Geneviève Corraze, Aires Oliva-Teles, Paula Enes

**Affiliations:** 1 CIIMAR—Interdisciplinary Centre of Marine and Environmental Research, University of Porto, Terminal de Cruzeiros do Porto de Leixões, Av. General Norton de Matos s/n, 4450-208 Matosinhos, Portugal; 2 Department of Biology, Faculty of Sciences, University of Porto, Rua do Campo Alegre s/n, Ed. FC4, 4169-007 Porto, Portugal; 3 Portuguese Institute for Sea and Atmosphere (IPMA), Olhão Pilot Aquaculture Station, Av. 5 de Outubro, s/n, 8700-305 Olhão, Portugal; 4 INRAE, University Pau & Pays Adour, E2S UPPA, UMR1419 NUMEA, 64310 Saint-Pee-sur-Nivelle, France

**Keywords:** *Argyrosomus regius*, glutathione, insect meal, lipid peroxidation, oxidative stress enzymes, *Tenebrio molitor*

## Abstract

This study aimed to determine the effects of dietary inclusion of *Tenebrio molitor* larvae (yellow worms) meal (TM) on meagre fish (*Argyrosomus regius*) whole-body fatty acids (FA) profile and hepatic and intestine oxidative status. For that purpose, fish were fed for 9 weeks a fishmeal-based diet (control) or diets including 10%, 20%, or 30% TM. With the increase in dietary TM level, whole-body oleic acid, linoleic acid, monounsaturated FA, and n−6 polyunsaturated FA (PUFA) increased while saturated FA (SFA), n−3 PUFA, n−3 long chain-PUFA, SFA:PUFA ratio, n3:n6 ratio, and FA retention decreased. Hepatic superoxide dismutase (SOD), glucose-6-phosphate dehydrogenase (G6PDH), and glutathione reductase (GR) activities increased and catalase (CAT) and glutathione peroxidase (GPX) activities decreased with dietary TM inclusion. Hepatic total and reduced glutathione were lower in fish fed 20% TM. Intestinal CAT activity and oxidized glutathione increased and GPX activity decreased with dietary TM inclusion. Intestine SOD, G6PDH, and GR activities increased and malondialdehyde concentration decreased in fish fed the diets with lower TM inclusion levels. Liver and intestine oxidative stress index and liver malondialdehyde concentration were unaffected by dietary TM. In conclusion, to avoid major whole-body FA changes or antioxidant status imbalances, it is recommended to limit TM to 10% inclusion in meagre diets.

## 1. Introduction

Yellow mealworm (*Tenebrio molitor*) (Tenebrionidae) has been on the list of insects that present the highest potential as food and feed in the European Union (EU) since 2015 [1]. In 2017, an EU Directive [2] authorized its use in aquafeeds, and in 2021, the European Food Safety Authority experts presented a favorable opinion for their use as a novel food for human nutrition [3]. *T. molitor* meal (TM) has a protein content of 45–60% (dry matter basis, DM) and a lipid content that ranges between 20 and 43% DM. Both larvae and pupae have an adequate amino acid profile, although low in sulfur amino acid content, and their fatty acids (FA) profile varies depending on the insect life stage, rearing environment, rearing substrate, and biomass processing methods [4,5,6]. TM lipid content is rich in oleic acid (38–50% total FA), linoleic acid (22–32% total FA), and palmitic acid (18–20% total FA) [5,7].

Fish whole-body FA composition is, at least, partially modulated by dietary FA, and previous studies observed that the use of insect meal in the diets affected fish FA composition [8,9,10,11]. The high unsaturated FA level of TM may lead to an increase in whole-body polyunsaturated FA (PUFA), and thus increase susceptibility to oxidation since oxygen radicals attack the double bonds present in PUFA [12,13,14].

On the other hand, TM may positively affect fish’s oxidative status, as *T. molitor* larvae lipids are rich in monounsaturated FA (MUFA) and PUFA which are known to have anti-inflammatory properties. In addition, *T. molitor* lipids are also rich in bioactive nutrients such as tocopherol, namely, ɣ-tocopherol, a primary lipid-soluble antioxidant [4,12]. Other TM components with antioxidant activity are chitin and chitosan, which have free radical-scavenging activities [4,5,15].

Several studies reported increased antioxidant potential or decreased oxidative damage in fish fed with diets including TM. For instance, yellow catfish (*Pelteobagrus fulvidraco*) fed diets with 9, 18, and 27% TM presented decreased plasma malondialdehyde (MDA) content, a marker of lipid peroxidation, and increased plasma superoxide dismutase (SOD) activity in fish fed with 9% and 27% TM [16]. Rainbow trout (*Oncorhynchus mykiss*) fed diets with 25 and 50% full-fat TM presented decreased MDA content and increased SOD, catalase (CAT), glutathione peroxidase (GPX), glutathione reductase (GR), and glucose-6-phosphate dehydrogenase (G6PDH) activities in the proximal intestine [17]. Contradictory results were also observed in some studies. For instance, tench (*Tinca tinca*) fed diets with 5.1% and 10.7% full-fat TM presented decreased intestine MDA content and SOD activity, and increased CAT activity in fish fed 10.7% TM [18]. Largemouth bass (*Micropterus salmoides*) fed diets with increasing levels of defatted TM (between 4% and 24.5%) showed decreased intestine MDA content (4, 16.3, 20.4, and 24.5%) and SOD activity (12.2%), but increased CAT activity (4%) and glutathione content (16.3 and 20.4%) [19]. Additionally, pearl gentian grouper (*Epinephelus lanceolatus* ♂ x *Epinephelus fuscoguttatus* ♀) fed diets with defatted TM (between 5% and 12.5%) presented increased liver MDA content (5%, 10%, and 12.5%) and decreased liver SOD activity (7.5%) [20]. In European sea bass (*Dicentrarchus labrax*) fed 50% full-fat TM, there was an increase in SOD (heart, muscle, and intestine), CAT (intestine), and GR (muscle and intestine) activities, heat shock proteins 70 and 90 (muscle), and of the apoptotic and autophagic machinery, which indicated a low tolerance to TM in this species [21].

Except for the study of Sankian et al. [8] in mandarin fish (*Siniperca scherzeri*) fed 10, 20, and 30% full-fat TM, no other studies have simultaneously assessed the effect of diets including insect meals on fish FA composition and oxidative stress markers. Authors found that fillets of fish fed TM had increased levels of saturated FA (SFA) and MUFA, and lower levels of n−3 PUFA. Serum SOD activity was not affected, and GPX activity increased with TM dietary increase [8]. However, authors only measured SOD and GPX activities and did not report the effects on the lipid peroxidation level or non-enzymatic antioxidant response. Thus, the present study aimed to determine the effects of diets including TM on whole-body FA profile, liver (the main metabolic tissue) and intestine (the tissue that has a direct contact with the feed components) enzymatic and non-enzymatic antioxidant response, and lipid peroxidation susceptibility of meagre (*Argyrosomus regius*) juveniles, a species of interest for Mediterranean aquaculture.

## 2. Materials and Methods

### 2.1. Experimental Diets and Growth Trial

Four isoproteic (50%) and isolipidic (19%) experimental diets were formulated as described in Coutinho et al. [22]. Shortly, a fishmeal-based (40% fishmeal) diet was used as a control (CTR diet), and three other diets were formulated to include 10%, 20%, and 30% of partially defatted yellow mealworm (*Tenebrio molitor*) meal (TM) (diets TM10, TM20 and TM30, respectively) replacing 25%, 50%, and 75% of fishmeal, respectively. *T. molitor* larvae were reared on a vegetal substrate. After harvesting, larvae were sieved to separate from the substrate, killed by heat, dried, and partially defatted by a cold screw press. The press cake was then hammer-milled. Fish oil was used as the main lipid source. All dietary ingredients were finely ground, well mixed and dry pelleted in a laboratory pellet mill (California Pellet Mill, CPM Crawfordsville, IN, USA) through a 2 mm die. The pellets were dried in an oven at 40 °C for 24 h and stored at −20 °C in airtight bags until use [22]. TM proximate analysis (74% protein, 13% lipids, 4.8% chitin) and amino acid composition, and experimental diets amino acid composition are presented in Coutinho et al. [22]. Experimental diets ingredient composition and proximate analysis are presented in Table 1.

The growth trial was performed with triplicate groups of meagre (*Argyrosomus regius*) juveniles with an initial mean body weight of 18.0 ± 0.02 g. The fish were fed by hand until visual satiation (utmost care was taken to avoid waste and to assure that all feed was consumed), twice a day, 6 days per week, for 9 weeks, as described in Coutinho et al. [22].

The growth trial was conducted according to the European Union Directive (2010/63/EU) on the protection of animals for scientific purposes, approved by the General Directorate of Food and Veterinary from Portugal (Certification number ORBEA-CIIMAR 30-2019), and directed by accredited scientists (following FELASA category C recommendations).

### 2.2. Sampling and Proximate Analysis

At the end of the trial, 9 fish from each tank were randomly sampled 5 h after the morning meal and sacrificed with a sharp blow to the head. Six fish were dissected on chilled trays for liver and whole-intestine collection (3 fish/tank for enzymatic activity and MDA concentration determination, and 3 fish/tank for glutathione measurement). After collection, the liver and intestine were immediately frozen in liquid nitrogen and stored at −80 °C until analysis. The remaining 3 fish/tank were pooled and stored at −80 °C for whole-body FA composition analyses. For FA composition analyses, total lipids were extracted and measured gravimetrically according to Folch et al. [23], using dichloromethane instead of chloroform. FA methyl esters were prepared according to Santha and Ackman [24] and were analyzed in a Varian 3900 gas chromatograph as described by Castro et al. [25].

### 2.3. Enzymes Activities, Lipid Peroxidation Determination, and Glutathione

Samples of liver (dilution 1:7) and intestine (dilution 1:5) were homogenized in ice-cold buffer (100 mM Tris-HCL, 0.1 mM EDTA, 0.1% Triton X-100 (*v*/*v*), pH 7.8). Homogenates were centrifuged at 30,000× *g* for 30 min at 4°C, aliquots of the resultant supernatant were collected and stored at −80 °C until analysis.

Superoxide dismutase (SOD; EC 1.15.1.1) activity was measured according to McCord and Fridovich [26], catalase (CAT; EC 1.11.1.6) activity according to Aebi [27], glucose-6-phosphate dehydrogenase (G6PDH; EC 1.1.1.49) activity according to Morales et al. [28], glutathione reductase (GR; EC 1.6.4.2) activity according to Morales et al. [29], and glutathione peroxidase (GPX; EC 1.11.1.9) activity according to Flohé and Günzler [30]. Protein concentration in liver and intestine homogenates was determined according to Bradford [31] using Bio-Rad Protein Assay Dye Reagent (ref. 5,000,006, Amadora, Portugal) with bovine serum albumin as standard. All enzymatic assays were performed at 37 °C in a Multiskan GO Microplate Reader (Model 5111 9200; Thermo Scientific, Nanjing, China).

MDA concentration was used as a marker of lipid peroxidation and was measured according to Buege and Aust [32]. Values were expressed as nmol MDA per g of wet tissue, calculated from a calibration curve.

Total glutathione (tGSH) and oxidized glutathione (GSSG) were measured following the methods described by Griffith [33] and Vandeputte et al. [34] with modifications as described by Castro et al. [35]. Reduced glutathione (GSH) was calculated by the difference between tGSH and GSSG values.

### 2.4. Statistical Analysis

Before being analyzed by one-way ANOVA, all data were verified for normal distribution by the Shapiro–Wilk test and homogeneity of variances by Levene’s test and normalized when appropriate. To determine the response to dietary TM inclusion, polynomial contrasts were performed to assess whether the data followed a linear or a quadratic response. For rejection of the null hypothesis, a significant level of 0.05 was used. Tukey’s multiple range tests were applied after ANOVA when *p* < 0.05, to illustrate the magnitude of the differences between means. All statistical analysis was performed using SPSS 24.0 software package for Windows (IBM^®^ SPSS^®^ Statistics, New York, NY, USA).

## 3. Results

Fish growth performance was not the goal of the present study and data are presented elsewhere [22]. In brief, fish final weight (80.5 g CTR, 66.1 g TM10, 53.2 g TM20, 40.0 g TM30), feed efficiency (1.25 CTR, 1.11 TM10, 1.03 TM20, 0.83 TM30), protein efficiency ratio (2.49 CTR, 2.22 TM10, 2.07 TM20, 1.67 TM30), and feed intake (16.3 g kg ABM^−1^ day^−1^ CTR, 16.6 g kg ABM^−1^ day^−1^ TM10, 15.5 g kg ABM^−1^ day^−1^ TM20, 14.5 g kg ABM^−1^ day^−1^ TM30; ABM: average body weight) linearly decreased with TM dietary inclusion level. Moreover, lipid whole-body composition (6.0% CTR, 5.5% TM10, 5.4% TM20, 5.1% TM30) also decreased with TM dietary inclusion level.

Oleic acid (18:1, 35.2%), linoleic acid (18:2n−6, 28.7%), and palmitic acid (16:0, 20.1%) were the most abundant FA in TM, with all the others representing less than 5% of total FA (Table 2). Overall, MUFA (38.7%) were the most abundant FA, followed by SFA (29.2%) and n−6 PUFA (28.7%), while n−3 PUFA represented only 1.17% of the total FA. Arachidonic acid (ARA, 20:4n−6), eicosapentaenoic acid (EPA, 20:5n−3), and docosahexaenoic acid (DHA, 22:6n−3) were not detected. Thus, with the increase in TM incorporation, dietary oleic acid, linoleic acid, and total MUFA increased, while palmitic acid, total SFA, and total n−3 PUFA decreased. SFA:PUFA and n3:n6 ratios, and the unsaturation index also decreased as dietary inclusion of TM increased.

The whole-body FA profile of meagre was affected by TM dietary inclusion (Table 3). Thus, with the increase in dietary TM level, there was a linear increase in MUFA and n−6 PUFA, mainly due to the increase in oleic and linoleic acids, and a linear decrease in SFA, n−3 PUFA, n−3 LC-PUFA, and of the SFA:PUFA, and n3:n6 ratios. Whole-body lipid and all the measured FA retention also linearly decreased with dietary TM inclusion (Table 4).

In the liver, SOD, G6PDH, and GR activities linearly increased while CAT and GPX activities linearly decreased with dietary TM inclusion level (Table 5). The tGSH and GSH decreased in fish fed the diets with lower TM inclusion levels and increased up to the control level in fish fed with diet TM30. GSSG, oxidative stress index (OSI), and MDA levels were not affected by dietary TM inclusion.

Intestine SOD, G6PDH, and GR activities increased in fish fed the diets with lower TM inclusion levels, and decreased up to the control level in fish fed with diet TM30 (Table 6). CAT activity linearly increased and GPX activity linearly decreased with dietary TM inclusion level. GSSG linearly increased with dietary TM inclusion, while tGSH, GSH, and OSI were not affected by the inclusion of TM. MDA level decreased in fish fed the diets with lower TM inclusion levels and increased up to the control level in fish fed with diet TM30.

## 4. Discussion

TM used in the present study had a lipid content of 13% (DM) and dietary inclusion of TM meal in the diets led to a contribution of up to 3.9% of TM oil in the diets, replacing a similar quantity of fish oil to keep diets isolipidic. Thus, TM oil amounted to up to 20% of total dietary lipids in the diets.

It is known that fish susceptibility to oxidative stress is affected by their FA composition [14] which, at least partially, is shaped by dietary FA composition [13]. The results of the present study showed that meagre whole-body FA profile and liver and intestine antioxidant status were highly affected by TM oil in the diets.

The most abundant FA in the partially defatted TM used in this study were oleic acid (18:1), linoleic acid (18:2n−6), and palmitic acid (16:0), which are the FA also reported in other studies with TM, indicating that regardless of the source and rearing conditions, these seem to be the most representative FA in TM oils [8,36,37,38,39]. Differences in the relative abundance of FA may, however, occur due to the insect life stage, rearing environment, and substrate used [4,5].

In the present study, MUFA were the most abundant FA followed by SFA and n−6 PUFA. Belforti et al. [36], Gasco et al. [37], and Sánchez-Muros et al. [38], also reported MUFA as the most abundant FA, followed by PUFA and SFA. On the other hand, Iaconisi et al. [39] reported n−6 PUFA as the most abundant FA, followed by MUFA and SFA.

Meagre whole-body FA profile resembled the dietary FA profile, with oleic acid, linoleic acid, MUFA, and n−6 PUFA linearly increasing with the dietary inclusion of TM. As TM lipids did not contain ARA, EPA, and DHA, the dietary level of these essential FA decreased as dietary TM was included in the diets, due to the decrease in dietary fish oil content. Similarly, an increase in oleic acid, linoleic acid, and n−6 PUFA and a decrease in EPA, DHA, and n−3 PUFA were also observed in rainbow trout muscle and European sea bass whole-body with the increase in dietary TM inclusion [36,37].

Together with EPA and DHA contents, the n3:n6 ratio is considered a good indicator of the nutritional value of fish as food, and the decrease of whole-body n3:n6 ratio with the dietary inclusion of TM suggests that the nutritional value of meagre fillets may be negatively affected by the use of TM as also suggested for blackspot seabream (*Pagellus bogaraveo*) [39].

In the present study, and although TM has a high palmitic acid content, the level of this FA decreased in the diets with TM increase, which was also observed in meagre whole-body FA composition. Fishmeal is richer in palmitic acid than TM [38], thus explaining the decrease in this FA as the fishmeal dietary inclusion level decreased.

With the increase in dietary TM, whole-body lipid and FA retention decreased, as a percentage of the intake, which may be related in part to the decrease in feed intake observed in meagre fed with diets including TM [22]. Although it is considered that fish spare EPA and DHA and that, therefore, their retention increases as the dietary level decreases, this was not observed in the present study.

The whole-body SFA:PUFA ratio linearly decreased with dietary TM increase, mainly as a result of the linear increase in linoleic acid, a PUFA, with TM increase. Thus, with the increase in PUFA level, more susceptibility to oxidation was expected to occur, since oxygen radicals attack more easily the double bonds present in PUFA [12,14]. However, it seems that in meagre the peroxidation levels (MDA) were not affected by dietary TM lipid composition, since despite some correlations (all with Pearson correlation coefficients (r) < 0.4) that were observed, none had statistical significance (data not shown).

To avoid oxidative damage to the cells, a set of antioxidant enzymes, such as SOD, CAT, GPX, and GR, and non-enzymatic antioxidants, such as glutathione, work together as part of the fish antioxidant defense system. SOD catalyzes the dismutation of superoxide anion (O^2−^) to molecular oxygen (O_2_) and hydrogen peroxide (H_2_O_2_), being the first enzyme responding to the presence of oxygen radicals, and CAT and GPX reduce the H_2_O_2_ produced to O_2_ and water (H_2_O) [40]. Glutathione can directly scavenge reactive oxygen species and is also required for the reduction of H_2_O_2_ to H_2_O by GPX. The maintenance of the reduced to oxidized glutathione (GSH/GSSG) ratio is achieved by the continuous reduction of GSSG to GSH by GR, which requires NADPH, which is mainly provided by G6PDH activity [41,42].

In the present study, a linear increase was observed in liver SOD, G6PDH, and GR activities and intestinal CAT activity in meagre fed with diets including increasing TM levels, which could indicate a higher antioxidant potential in fish fed increasing TM levels. Similarly, yellow catfish fed TM presented increased plasma SOD activity [16], and rainbow trout presented increased proximal intestine SOD, G6PDH, and GR activities [17]. In these studies, also observed was a decrease in the plasma [16] and proximal intestine [17] MDA content with the increase in dietary TM inclusion while in the present study, the liver MDA content was not affected by the dietary treatments, and in the intestine, a decrease was observed only in meagre fed diets TM10 and TM20 but not TM30. Overall, it seems that fish were capable of maintaining their oxidative status independently of diet composition.

The response of the antioxidant defense mechanisms was tissue-dependent. Thus, intestine showed a higher MDA content compared with liver, which is possibly related with the higher susceptibility of intestine to oxidative stress, due to the high cell turnover and direct contact with the feed components. Accordingly, SOD, the first enzyme responding to the presence of oxygen radicals, presented increased activity in the intestine. GR activity and GSSG content were also higher in intestine, while CAT, G6PDH, and GPX activities were higher in liver. Higher CAT, G6PDH, and GPX activities in liver than in intestine were also reported in gilthead seabream (*Sparus aurata*) and European sea bass [35,43]. Another study in gilthead seabream also observed higher SOD activity in intestine as in the present study [44]. Those results support that fish antioxidant system responds differently depending on the analyzed tissue. Moreover, the liver is the main producer and supplier of GSH, while the intestine presents a limited capacity for GSH synthesis or accumulation, despite being a major consumer of GSH [41,45]. Thus, a higher GSH in the liver was already expected. Similarly, lower GSH content was observed in the intestine of gilthead seabream and European sea bass when compared with the liver [35,43,44].

GPX activity decreased in both tissues with the increase in dietary inclusion of TM and this can be related to the decrease of arginine and lysine in the TM diets compared to the control [22]. Since the binding site of GSH to GPX contains one lysine and four arginine residues [46], the decrease of those amino acids in the TM-based diets might have contributed to reducing GPX efficiency.

In the intestine, the decrease in GPX activity was compensated by an increase in CAT activity, while that relationship was not observed in the liver. Indeed, in the liver, CAT activity decreased with the increase in dietary TM inclusion. This apparent contradictory response in the two tissues may be related to the level of oxidative stress they were exposed to, and to the basal level of activity of the majority of the oxidative stress enzymes, which was much higher in the liver than in the intestine. As a consequence, the MDA levels in the liver were much lower than in the intestine, indicating that oxidative status was more controlled in the liver than in the intestine. Similarly, in studies with, for instance, gilthead seabream and European sea bass, higher MDA content was observed in the intestine when compared with the liver [35,43,44].

## 5. Conclusions

In conclusion, feeding meagre with diets including TM highly affected the whole-body FA profile. While the overall liver and intestinal oxidative status of the animals was not affected, the response mechanisms to the oxidative stress were affected by the dietary inclusion of TM. Thus, based on the present results, and to avoid major whole-body FA profile changes and antioxidant status imbalances, it is not recommended to incorporate more than 10% TM replacing 25% fishmeal in meagre diets. This suggestion is in line with previous results which showed that even the dietary inclusion of 10% of partially defatted TM lead to a decreased digestive capacity and growth performance, and that higher inclusion levels also negatively affect fish whole-body composition, with authors concluding that meagre has a limited capacity to utilize TM [22].

## Figures and Tables

**Table 1:** Ingredient composition and proximate analysis of the experimental diets [22].

	Diets			
	CTR	HM10	HM20	HM30
*Ingredients (% dry weight basis)*				
Fish meal ^1^	40.0	30.1	20.1	10.2
Soluble fish protein concentrate ^2^	2.5	2.5	2.5	2.5
*Tenebrio molitor * ^3^	-	10.0	20.0	30.0
Wheat gluten ^4^	5.0	5.0	5.0	5.0
Corn gluten ^5^	7.5	7.5	7.5	7.5
Soybean meal ^6^	14.0	14.0	14.0	14.0
Wheat meal ^7^	15.0	14.3	13.6	12.8
Fish oil	12.3	12.1	12.0	11.8
Vitamin premix ^8^	1.0	1.0	1.0	1.0
Mineral premix ^9^	1.0	1.0	1.0	1.0
Choline chloride (50%)	0.5	0.5	0.5	0.5
Binder ^10^	1.0	1.0	1.0	1.0
Taurine ^11^	0.2	0.2	0.2	0.2
Dibasic calcium phosphate	-	0.8	1.6	2.5
*Proximate analyses (% dry weight basis)*				
Dry matter	93.7	91.8	94.3	90.8
Crude protein	50.3	50.1	49.7	49.7
Crude fat	19.0	18.7	19.2	19.9
Ash	9.2	8.8	8.5	8.5
Energy (kJ g^−1^)	23.2	23.6	23.1	23.9
Chitin	0.0	0.74	0.97	1.47

CP: crude protein; DM: dry matter; GL: gross lipid.^1^ Steam-Dried LT-FM, Copicesa S. A., Spain(CP: 73.2% DM; GL: 11.4% DM).^2^ Sopropèche G, France (CP: 77.0% DM; GL: 18.4% DM).^3^ HiProMine S.A., Robakowo, Poland.^4^ Sorgal, S.A. Ovar, Portugal (CP: 83.1% DM; CL: 1.9% DM).^5^ Sorgal, S.A. Ovar, Portugal (CP: 70.2% DM; CL: 2.3% DM).^6^ Sorgal, S.A. Ovar, Portugal (CP: 50.6% DM; CL: 1.6% DM).^7^ Sorgal, S.A. Ovar, Portugal (CP: 14.3% DM; CL: 2.0% DM).^8^ Vitamins (mg kg^−1^ diet): retinol, 18,000 (IU); cholecalciferol, 2000 (IU); α-tocopherol, 35; menadione sodium bisulphate, 10; thiamine, 15; riboflavin, 25; Ca pantothenate, 50; nicotinic acid, 200; pyridoxine, 5; folic acid, 10; cyanocobalamin, 0.02; biotin, 1.5; ascorbyl monophosphate, 50; inositol, 400.^9^ Minerals (mg kg^−1^ diet): cobalt sulphate, 1.91; copper sulphate, 19.6; iron sulphate, 200; sodium fluoride, 2.21; potassium iodide, 0.78; magnesium oxide, 830; manganese oxide, 26; sodium selenite, 0.66; zinc oxide, 37.5; dibasic calcium phosphate, 5.93; potassium chloride, 1.15; sodium chloride, 0.44.^10^ Aquacube. Agil, UK.^11^ Feed-grade taurine, Sorgal, S.A. Ovar, Portugal.

**Table 2:** Fatty acid composition (% of total fatty acids) of *Tenebrio molitor* and of the experimental diets fed to meagre.

	Insect Meal	Diets			
Fatty Acids	*Tenebrio Molitor*	CTR	TM10	TM20	TM30
12:0	0.48	0.12	0.22	0.21	0.24
14:0	4.10	8.61	7.79	7.72	7.40
15:0	0.38	0.88	0.79	0.70	0.68
16:0	20.1	25.3	24.3	24.2	23.5
17:0	0.36	0.49	0.49	0.46	0.43
18:0	3.74	3.16	3.24	3.16	3.11
ΣSFA	29.2	38.6	36.8	36.4	35.4
14:1	0.18	0.33	0.29	0.27	0.26
16:1	3.37	8.34	7.62	7.38	6.82
17:1	0.00	0.15	0.15	0.06	0.05
18:1	35.2	22.7	24.3	26.1	27.3
20:1	0.00	2.27	2.31	1.64	1.67
22:1	0.00	1.17	1.27	1.07	0.87
ΣMUFA	38.7	35.0	35.9	36.5	36.9
18:2 n−6	28.7	8.77	11.2	13.6	15.5
20:2 n−6	0.00	0.21	0.21	0.15	0.10
20:4 n−6	0.00	0.45	0.42	0.29	0.14
Σn−6 PUFA	28.7	9.43	11.9	14.0	15.8
18:3 n−3	1.17	2.26	2.20	2.16	1.99
18:4 n−3	0.00	2.23	1.99	1.73	1.52
20:4 n−3	0.00	0.30	0.21	0.00	0.15
20:5 n−3	0.00	4.51	4.16	3.65	3.16
22:6 n−3	0.00	3.57	3.44	2.77	2.30
Σn−3 PUFA	1.17	12.9	12.0	10.31	9.11
Σn−3 LC-PUFA	0.00	8.39	7.81	6.42	5.61
Ratios					
SFA:PUFA	0.96	1.58	1.43	1.39	1.33
n3:n6	0.04	1.37	1.01	0.74	0.58
Unsaturation Index ^a^	101.0	122.5	123.0	118.8	116.2

LC-PUFA, long chain-polyunsaturated fatty acids; MUFA, monounsaturated fatty acids; SFA, saturated fatty acids. Fatty acids ≥ 0.02%, when <0.02% were not considered in the table as it was below detection. ^a^ Unsaturation Index = sum (fatty acid %) × (number of double bonds).

**Table 3:** Whole-body fatty acid profile (% of total fatty acids) of meagre before the beginning of the trial and fed the experimental diets.

		Diets				One-Way ANOVA	Polynomial Contrasts	
Fatty Acids	Initial	CTR	TM10	TM20	TM30	*p*-Value	Linear	Quadratic
14:0	3.8	6.20 ± 0.25 ^b^	5.93 ± 0.63 ^ab^	5.39 ± 0.24 ^ab^	4.98 ± 0.19 ^a^	0.016	0.002	0.749
15:0	0.5	0.78 ± 0.032	0.57 ± 0.339	0.68 ± 0.042	0.61 ± 0.012	0.483	0.364	0.501
16:0	24.3	25.3 ± 1.09	24.6 ± 1.94	23.0 ± 0.47	22.5 ± 0.46	0.058	0.011	0.838
17:0	0.4	0.49 ± 0.04 ^b^	0.41 ± 0.024 ^ab^	0.39 ± 0.025 ^a^	0.38 ± 0.013 ^a^	0.007	0.002	0.086
18:0	4.8	3.43 ± 0.11 ^b^	3.01 ± 0.14 ^a^	3.12 ± 0.10 ^a^	3.18 ± 0.09 ^ab^	0.010	0.056	0.006
ΣSFA	33.9	36.2 ± 1.38 ^b^	34.5 ± 2.77 ^ab^	32.6 ± 0.61 ^ab^	31.7 ± 0.65 ^a^	0.035	0.006	0.673
14:1	0.1	0.25 ± 0.022 ^b^	0.23 ± 0.033 ^ab^	0.20 ± 0.025 ^ab^	0.17 ± 0.018 ^a^	0.016	0.002	0.687
16:1	7.5	8.07 ± 0.44	8.38 ± 0.31	8.06 ± 0.09	7.73 ± 0.32	0.183	0.147	0.118
17:1	0.0	0.16 ± 0.018 ^b^	0.13 ± 0.035 ^ab^	0.09 ± 0.007 ^a^	0.12 ± 0.003 ^ab^	0.011	0.008	0.020
18:1	28.2	24.1 ± 0.65 ^a^	25.3 ± 0.31 ^b^	27.0 ± 0.34 ^c^	29.1 ± 0.24 ^d^	0.000	0.000	0.114
20:1	1.2	2.34 ± 0.17 ^b^	2.06 ± 0.31 ^ab^	1.94 ± 0.13 ^ab^	1.72 ± 0.04 ^a^	0.024	0.004	0.802
22:1	0.5	0.66 ± 0.481	0.89 ± 0.226	0.88 ± 0.078	0.67 ± 0.085	0.600	0.993	0.198
ΣMUFA	37.5	35.5 ± 1.07 ^a^	37 ± 0.54 ^ab^	38.2 ± 0.21 ^bc^	39.5 ± 0.21 ^c^	0.000	0.000	0.868
18:2n−6	14.9	10.5 ± 0.28 ^a^	12.5 ± 0.13 ^b^	14.9 ± 0.42 ^c^	16.8 ± 0.3 ^d^	0.000	0.000	0.779
20:2n−6	0.0	0.23 ± 0.021 ^ab^	0.24 ± 0.018 ^b^	0.19 ± 0.022 ^a^	0.19 ± 0.02 ^ab^	0.023	0.010	0.954
20:4n−6	0.8	0.67 ± 0.025	0.55 ± 0.164	0.50 ± 0.088	0.45 ± 0.032	0.102	0.021	0.571
Σn−6 PUFA	15.8	11.4 ± 0.28 ^a^	13.3 ± 0.31 ^b^	15.6 ± 0.42 ^c^	17.5 ± 0.29 ^d^	0.000	0.000	0.925
18:3n−3	1.8	2.03 ± 0.082 ^c^	1.88 ± 0.026 ^b^	1.86 ± 0.035 ^ab^	1.73 ± 0.043 ^a^	0.001	0.000	0.693
18:4n−3	0.7	1.56 ± 0.076 ^d^	1.35 ± 0.042 ^c^	1.17 ± 0.025 ^b^	0.98 ± 0.045 ^a^	0.000	0.000	0.736
20:4n−3	0.2	0.33 ± 0.058 ^b^	0.26 ± 0.036 ^ab^	0.21 ± 0.035 ^a^	0.19 ± 0.012 ^a^	0.011	0.002	0.281
20:5n−3	2.7	3.94 ± 0.38 ^c^	3.34 ± 0.29 ^bc^	3.04 ± 0.26 ^ab^	2.50 ± 0.2 ^a^	0.002	0.000	0.851
22:6n−3	3.1	4.98 ± 0.9 ^b^	4.09 ± 0.71 ^ab^	3.72 ± 0.16 ^ab^	2.74 ± 0.28 ^a^	0.011	0.002	0.894
Σn−3 PUFA	8.5	12.8 ± 1.37 ^c^	10.9 ± 0.95 ^bc^	10 ± 0.44 ^ab^	8.1 ± 0.41 ^a^	0.001	0.000	0.950
Σn−3 LC-PUFA	6.0	9.2 ± 1.33 ^b^	7.7 ± 0.10 ^ab^	7.0 ± 0.42 ^ab^	5.4 ± 0.49 ^a^	0.005	0.001	0.983
Ratios								
SFA:PUFA	1.31	1.37 ± 0.11	1.31 ± 0.16	1.18 ± 0.04	1.16 ± 0.02	0.091	0.018	0.743
n3:n6	0.54	1.13 ± 0.136 ^c^	0.82 ± 0.054 ^b^	0.64 ± 0.034 ^ab^	0.47 ± 0.036 ^a^	0.000	0.000	0.172
Unsaturation Index ^1^	116.4	128.5 ± 6.33	123.7 ± 6.69	124.1 ± 2.32	118.8 ± 1.89	0.188	0.051	0.925

Mean values and standard deviation (±SD) are presented for each parameter (*n* = 3, 1 pool of 3 fish/tank). Different letters in the same row stand for statistical differences between diets (*p* < 0.05). ^1^ Unsaturation Index = sum (fatty acid %) × (number of double bonds). Fatty acids ≥ 0.02%, when <0.02% were not considered in the table as it was below detection. LC-PUFA, long chain-polyunsaturated fatty acids; MUFA, monounsaturated fatty acids; SFA, saturated fatty acids.

**Table 4:** Whole-body lipids retention (% lipids intake) and fatty acids retention (% fatty acid intake)^1^ of meagre fed the experimental diets.

	Diets				One-Way ANOVA	Polynomial Contrasts	
	CTR	TM10	TM20	TM30	*p*-Value	Linear	Quadratic
Lipids retention	47.1 ± 2.3 ^c^	41.0 ± 3.3 ^bc^	38.6 ± 3.3 ^b^	29.3 ± 2.7 ^a^	0.000	0.000	0.368
16:0	47.3 ± 3.3 ^c^	41.4 ± 1.4 ^bc^	36.5 ± 2.3 ^ab^	30.0 ± 4.4 ^a^	0.001	0.000	0.890
ΣSFA	44.5 ± 2.8 ^c^	38.4 ± 1.8 ^bc^	34.3 ± 2.2 ^ab^	28.1 ± 3.9 ^a^	0.001	0.000	0.962
18:1n−9	49.3 ± 3.8 ^b^	42.3 ± 4 ^ab^	39.7 ± 3.9 ^ab^	34.2 ± 5.7 ^a^	0.019	0.003	0.780
ΣMUFA	47.7 ± 3.8 ^b^	42.3 ± 4 ^ab^	40.4 ± 3.6 ^ab^	34.4 ± 5.4 ^a^	0.032	0.005	0.910
18:2n−6	54.6 ± 4.4 ^b^	44.8 ± 4.2 ^ab^	42.5 ± 4.8 ^ab^	35.4 ± 6.2 ^a^	0.010	0.002	0.654
Σn−6 PUFA	55.3 ± 4.4 ^b^	45.2 ± 4.9 ^ab^	43.0 ± 4.9 ^ab^	36.1 ± 6.3 ^a^	0.012	0.002	0.606
18:3n−3	42.7 ± 3.5 ^b^	35.3 ± 3 ^ab^	33.4 ± 3.4 ^ab^	27.6 ± 4.6 ^a^	0.007	0.001	0.705
20:5n−3	42.1 ± 3.9 ^b^	33.7 ± 5.6 ^ab^	32.7 ± 4.7 ^ab^	24.6 ± 2 ^a^	0.007	0.001	0.952
22:6n−3	67.3 ± 11.3 ^b^	50.3 ± 12.6 ^ab^	53.0 ± 3.7 ^ab^	36.6 ± 2.9 ^a^	0.018	0.004	0.949
Σn−3 PUFA	48.1 ± 4.9 ^b^	38.3 ± 6.3 ^ab^	38.2 ± 3.7 ^ab^	28.1 ± 2.6 ^a^	0.005	0.001	0.953

Mean values and standard deviation (±SD) are presented for each parameter (*n* = 3). Different letters in the same row stand for statistical differences between diets (*p* < 0.05). MUFA, monounsaturated fatty acids; PUFA, polyunsaturated fatty acids; SFA, saturated fatty acids. ^1^ Whole-body lipids or FA retention (% lipids or FA intake) = ((Final body weight × Final whole-body lipids or FA) − (Initial body weight × Initial whole-body lipids or FA))/(Feed intake x Dietary lipids or FA) × 100, where FA is fatty acids.

**Table 5:** Hepatic levels of superoxide dismutase (SOD), catalase (CAT) (U mg protein^−1^), glucose-6-phosphate dehydrogenase (G6PDH), glutathione reductase (GR), and glutathione peroxidase (GPX) (mU mg protein^−1^) activities and of total glutathione (tGSH), reduced glutathione (GSH), and oxidized glutathione (GSSG) (mmol g tissue^−1^), oxidative stress index (OSI, %), and malondialdehyde (MDA, nmol MDA g tissue^−1^) in meagre juveniles fed the experimental diets.

	Diets				One-Way ANOVA	Polynomial Contrasts	
	CTR	TM10	TM20	TM30	*p*-Value	Linear	Quadratic
SOD	91 ± 24 ^ab^	74 ± 18 ^a^	102 ± 14 ^bc^	119 ± 16 ^c^	0.000	0.000	0.014
CAT	320 ± 95 ^b^	325 ± 62 ^b^	278 ± 20 ^ab^	216 ± 42 ^a^	0.003	0.001	0.122
G6 PDH	72 ± 6 ^a^	82 ± 13 ^ab^	81 ± 10 ^ab^	94 ± 17 ^b^	0.010	0.002	0.668
GR	5.2 ± 0.51 ^a^	5.8 ± 1.16 ^ab^	6.0 ± 1.14 ^ab^	6.9 ± 1.34 ^b^	0.022	0.003	0.704
GPX	174 ± 35 ^c^	189 ± 29 ^c^	126 ± 23 ^b^	81 ± 24 ^a^	0.000	0.000	0.003
tGSH	1042 ± 91 ^b^	981 ± 130 ^b^	775 ± 119 ^a^	1007 ± 148 ^b^	0.000	0.130	0.002
GSH	1038 ± 91 ^b^	977 ± 129 ^b^	773 ± 118 ^a^	1002 ± 148 ^b^	0.000	0.126	0.002
GSSG	3.4 ± 1.48	4.4 ± 1.88	2.7 ± 1.31	4.5 ± 2.04	0.199	0.448	0.292
OSI ^1^	0.66 ± 0.30	0.8 ± 0.36	0.7 ± 0.30	0.9 ± 0.39	0.512	0.252	0.813
MDA	16 ± 5	20 ± 10	14 ± 8	26 ± 19	0.150	0.194	0.289

Mean values and standard deviation (± SD) are presented for each parameter (*n* = 9). Different letters in the same row stand for statistical differences between diets (*p* < 0.05). ^1^ OSI = 100 × (2 × GSSG/tGSH).

**Table 6:** Intestine levels of superoxide dismutase (SOD), catalase (CAT) (U mg protein^−1^), glucose-6-phosphate dehydrogenase (G6PDH), glutathione reductase (GR), and glutathione peroxidase (GPX) (mU mg protein^−1^) activities, and of total glutathione (tGSH), reduced glutathione (GSH), and oxidized glutathione (GSSG) (mmol g tissue^−1^), oxidative stress index (OSI, %), and malondialdehyde (MDA, nmol MDA g tissue^−1^) in meagre juveniles fed the experimental diets.

	Diets				One-Way ANOVA	Polynomial Contrasts	
	CTR	TM10	TM20	TM30	*p*-Value	Linear	Quadratic
SOD	333 ± 101	387 ± 130	370 ± 105	259 ± 37	0.065	0.140	0.023
CAT	46.8 ± 28.2 ^a^	71.5 ± 29.2 ^a^	67.8 ± 16.6 ^a^	101.4 ± 12.6 ^b^	0.000	0.000	0.567
G6PDH	3.28 ± 1.08	5.73 ± 2.56	4.92 ± 2.31	4.20 ± 2.33	0.116	0.547	0.034
GR	7.38 ± 1.92 ^a^	10.23 ± 2.72 ^b^	8.97 ± 1.31 ^ab^	7.19 ± 2.07 ^a^	0.011	0.558	0.002
GPX	43 ± 7.6 ^b^	42.2 ± 10.1 ^b^	40.3 ± 10.0 ^b^	25.4 ± 10.1 ^a^	0.002	0.001	0.042
tGSH	845 ± 189	768 ± 49	919 ± 301	877 ± 246	0.599	0.467	0.822
GSH	830 ± 189	747 ± 50	898 ± 299	858 ± 246	0.605	0.495	0.785
GSSG	14.7 ± 3.17 ^a^	16.8 ± 3.59 ^ab^	21.6 ± 6.83 ^b^	19.4 ± 4.25 ^ab^	0.026	0.013	0.205
OSI ^1^	3.67 ± 1.11	4.15 ± 1.11	4.45 ± 1.33	4.77 ± 1.73	0.379	0.088	0.866
MDA	102 ± 31	76 ± 30	69 ± 14	102 ± 37	0.041	0.921	0.005

Mean values and standard deviation (± SD) are presented for each parameter (*n*= 9). Different letters in the same row stand for statistical differences between diets (*p* < 0.05). ^1^ OSI = 100 × (2 × GSSG/tGSH).

## Data Availability

The data used to generate the results in this manuscript can be made available if requested from the corresponding author.
